# Interpreting forces as deep learning gradients improves quality of predicted protein structures

**DOI:** 10.1016/j.bpj.2023.12.011

**Published:** 2023-12-15

**Authors:** Jonathan Edward King, David Ryan Koes

**Affiliations:** 1 Joint PhD Program in Computational Biology, Carnegie Mellon University-University of Pittsburgh, Pittsburgh, Pennsylvania; 2 Computational & Systems Biology, University of Pittsburgh, Pittsburgh, Pennsylvania

## Abstract

Protein structure predictions from deep learning models like AlphaFold2, despite their remarkable accuracy, are likely insufficient for direct use in downstream tasks like molecular docking. The functionality of such models could be improved with a combination of increased accuracy and physical intuition. We propose a new method to train deep learning protein structure prediction models using molecular dynamics force fields to work toward these goals. Our custom PyTorch loss function, OpenMM-Loss, represents the potential energy of a predicted structure. OpenMM-Loss can be applied to any all-atom representation of a protein structure capable of mapping into our software package, SidechainNet. We demonstrate our method’s efficacy by finetuning OpenFold. We show that subsequently predicted protein structures, both before and after a relaxation procedure, exhibit comparable accuracy while displaying lower potential energy and improved structural quality as assessed by MolProbity metrics.

## Significance

We propose a novel framework to directly incorporate forces from molecular dynamics as gradients for training deep learning models like AlphaFold2. We implement our method as a PyTorch loss function, OpenMM-Loss, which frames the potential energy of predicted protein structures as a minimization objective. When applied to OpenFold, our method demonstrates improved structural quality, lower potential energy, and comparable accuracy relative to the OpenFold baseline. Our framework may enhance the ability of deep learning models to recapitulate fundamental biophysical principles, reducing the number of structural irregularities in their predictions and paving the way for more effective downstream applications.

## Introduction

Deep learning (DL) methods for protein structure prediction, particularly AlphaFold2 (AF2) (1) and AF2-like systems (2,3,4), have proven remarkably accurate. However, barriers to the practical use of AF2 predictions remain. Some of AF2’s shortcomings are due to limitations of its design, while others are simply areas where accuracy could be improved. For instance, Jumper et al. (1) trained AF2 on protein structures from the PDB (5). By virtue of their presence in the PDB, many of these proteins formed stable crystal structures in the lab. Such structures may not adequately reflect their in vivo conformations, which may exhibit higher energy under crystallization conditions. Another fundamental limitation of AF2 is its inability to simultaneously model proteins and their ligands. Without knowledge of protein-ligand interactions, AF2 cannot reliably predict *apo* or *holo* protein states, making drug development with these structures more challenging.

AF2 also likely needs higher accuracy for downstream tasks like docking. Several studies (6,7,8) have demonstrated that AF2-predicted structures are surprisingly nonperformant compared to crystal structures used for docking. Despite a relatively high level of accuracy of the protein backbone and binding sites, subtle differences between the prediction and crystal structure cause significantly different results when docking molecules. Side-chain atom accuracy is a likely culprit since differences in side-chain atom placement or orientation may dramatically alter the binding pocket shape or chemical environment. There have been several attempts to improve AF2 predictions in a postprocessing step, including using modified molecular dynamics (MD) simulations, induced-fit docking, and free energy perturbation analysis (9,10,11).

In addition to working toward more accurate models, the research community has expressed interest in developing models capable of understanding and recapitulating fundamental physical principles. Models that leverage existing scientific knowledge could free up computational resources to focus on subproblems better suited for machine learning instead of relearning principles taught in high school chemistry and mathematics. Such a model could, in principle, better guide drug discovery campaigns since it may better understand the chemical interactions between a protein and a ligand.

Researchers in several fields have tried utilizing more complex and physically relevant input representations and model architectures or even empirical data to improve prediction quality. For example, data representation in cheminformatics has shifted from SMILES (12) (string-based) representations of chemical structures to other representations like graphs (13,14), point clouds (15), or 3D voxels (16) that more closely resemble the physical reality of these systems. One DL method for computational drug discovery utilizes theoretical chemical principles in a Siamese neural network (17). By directly subtracting the latent spaces between the two component models, the authors model the relative free binding energy, ΔΔG, between two ligands, enforcing the symmetric properties of relative binding affinities, $\Delta \Delta G_{AB}=\Delta G_{A}-\Delta G_{B}=-\left(\Delta G_{B}-\Delta G_{A}\right)$. Active learning is also often used to incorporate experimental data into machine learning training procedures, guiding machine learning experiments with real-life data (18,19,20).

In the same spirit, AF2’s authors incorporate physiochemical constraints into their work by training with a “violation loss.” The violation loss is a component of AF2’s composite loss function that penalizes predictions for generating structures with overlapping (i.e., clashing) atoms or bond lengths and angles that deviate from the corresponding values in the literature. Despite the addition of the violation loss, AF2 predictions have significant stereochemical violations. The recommended prediction procedure involves a relaxation step with MD force fields (MD FFs), which takes several seconds but significantly improves the final structure by removing many of these violations. Triangle point attention, also developed by AF2’s authors, is suggested to provide physical and geometric intuition to AF2. However, because it is a neural network component with learned parameters, it is unclear how it works in practice or if it genuinely imbues the model with any physical constraints.

## Materials and methods

This section describes our method to improve structure predictions from methods like AF2 by incorporating physical principals from MD FFs. We will discuss the intuition for our approach, practical considerations for its development, and our procedure for training and evaluating our models with custom datasets.

OpenFold is an implementation of AF2 that attempts to faithfully reproduce AF2 in PyTorch with added utilities for training. OpenFold was developed by Ahdritz et al. (21) and AlphaFold2 by Jumper et al. (1). We refer to OpenFold and AlphaFold2 interchangeably as AF2, though we only work with the OpenFold code base.

### Forces from MD can be interpreted as gradients in backpropagation

To improve overall protein structure prediction accuracy and to help protein structure prediction models better understand physical principles, we propose capitalizing on the relationship between the physical forces computed by MD software (Eq. 1) and the gradients utilized in machine learning backpropagation (Eq. 2).

(1) $$F\left(X\right)=-\nabla_{X}U\left(X\right)$$

MD software evaluates the energy U(X) of a molecular system at each time step based on the positions of the atoms *X* and the FF parameters. Next, the software computes the forces acting on the atoms via Eq. 1. Since MD software can readily compute U(X) and $-\nabla_{X}U\left(X\right)$ for a protein system, we propose treating U(X) as a loss function whose gradients are −F(X).

(2) $$\begin{array}{l} \text{Loss}=U\left(X\right)\\ \nabla_{X}\left(\text{Loss}\right)=-F\left(X\right) \end{array}$$

For backpropagation machinery to utilize these gradients during training, all that is needed is a custom layer with “forward” and “backward” components that return U(X) and −F(X), respectively. We can then apply this framework to any machine learning model capable of predicting all-atom protein structure representations.

### OpenMM-based loss function

**Figure 1:**
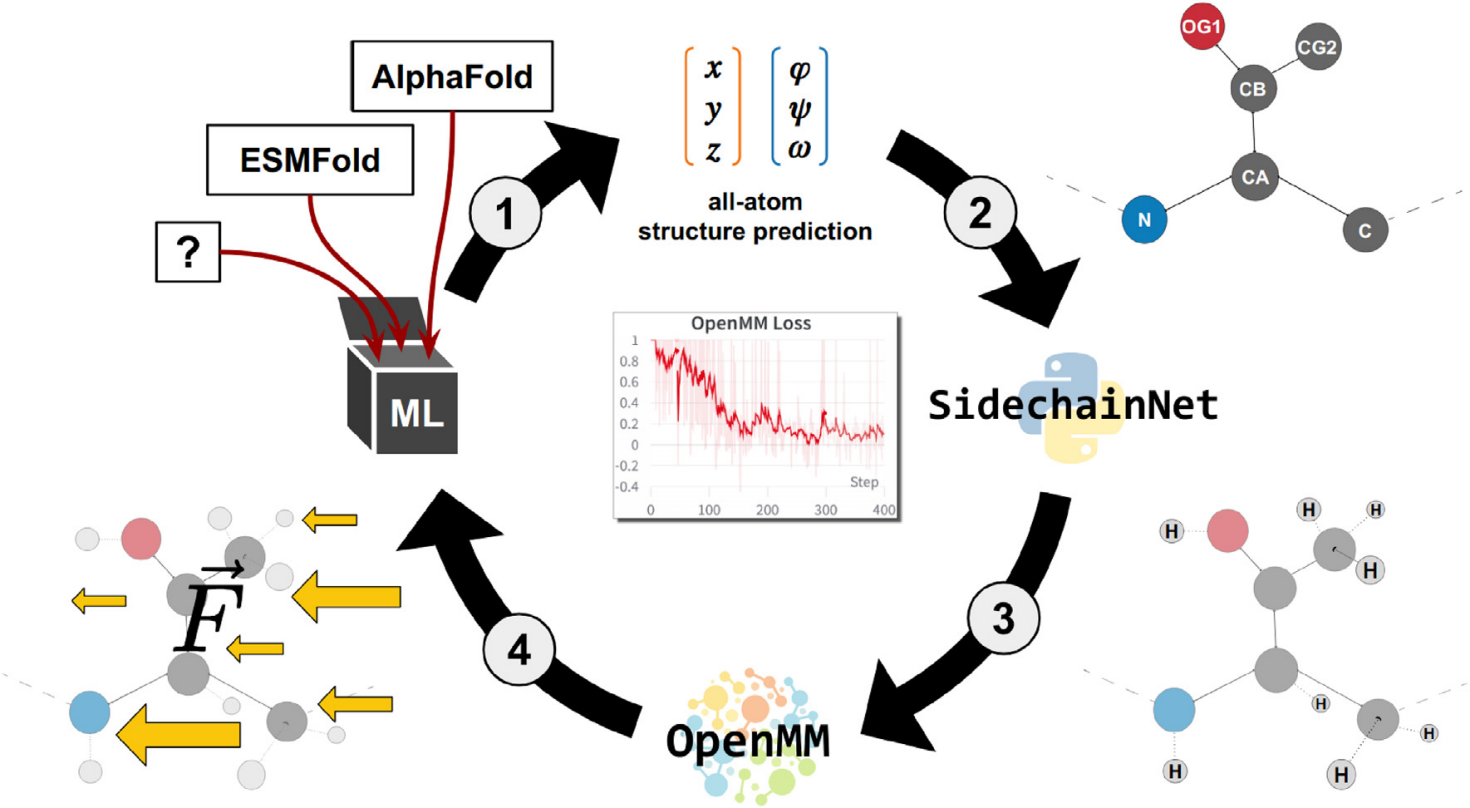
A graphical summary of our method for training deep learning protein structure prediction models by interpreting MD forces as gradients. To see this figure in color, go online.

Our custom loss function implements Eq. 2 via the steps depicted in Fig. 1. 1) A DL model predicts an all-atom protein structure representation. Our code supports Cartesian and internal coordinate (bond and torsional angle) representations of protein structure. 2) The user determines a mapping between the atoms or angles predicted by their model and the atom or angle representation expected by the SidechainNet protein representation. SidechainNet (22) is a Python library and dataset with tools for PyTorch-based (23) DL protein structure prediction tasks. 3) In preparation for energy evaluation with OpenMM (24), an MD system is initialized using the all-atom protein representation, and hydrogen atoms are added (if not already present) to the prediction using differentiable vector operations. 4) The custom loss function layer and SidechainNet protein representation compute a loss equal to the predicted structure’s potential energy. The forces acting on the atoms, also calculated by OpenMM, are designated as gradients to train the model. The model parameters are updated, and training continues.

**Figure 2:**
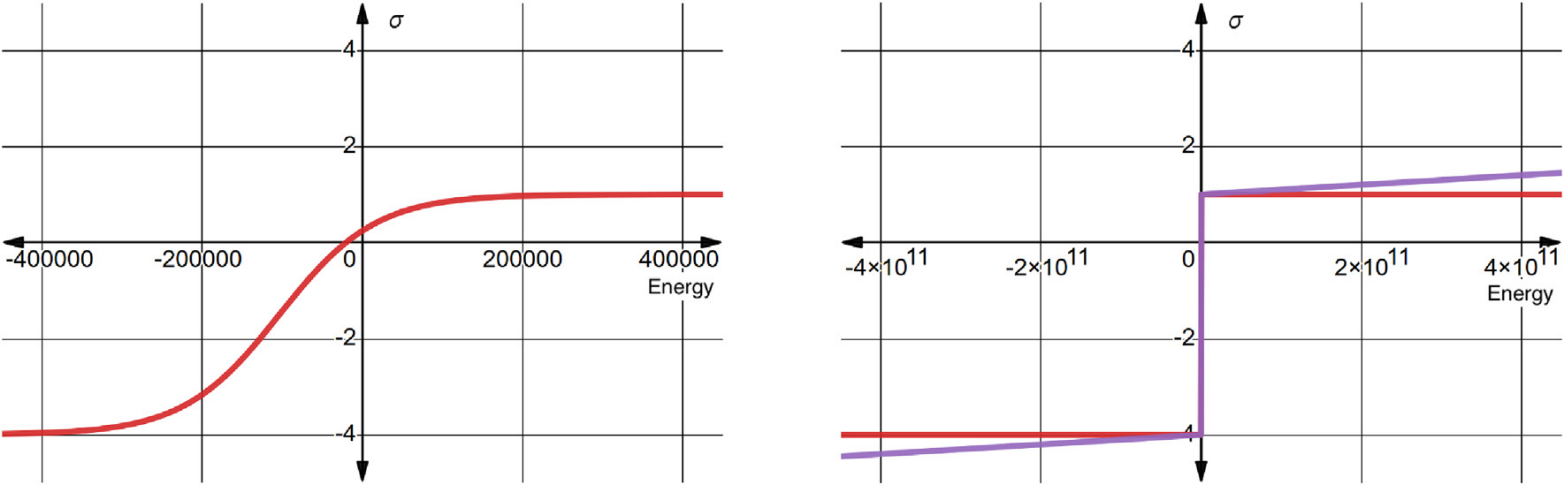
$\tilde{\sigma }$ and $\sigma^{*}$ in red and purple, respectively. Both functions have nearly identical behavior, except $\sigma^{*}$ has a nonzero derivative for extreme values of *x* (energy). To see this figure in color, go online.

Users may specify any FF and solvent model available in the OpenMM package for use with our method. For experiments in this article, we selected the generalized Born implicit solvent model along with the ff15ipq FF (25).

#### Practical consideration I: Hydrogens

Let $X_{H}$ and $X_{\bar{H}}$ represent predicted protein structures with and without hydrogen atoms, respectively. MD FFs require protein systems to contain hydrogen atoms in order to compute $U\left(X_{H}\right)$ and $F\left(X_{H}\right)$. However, AF2 and related models predict structures without hydrogens, $X_{\bar{H}}$. Thus, we must find a way to add hydrogens to the structures predicted by these models during training so that we may utilize the values computed by OpenMM. If hydrogens are added to the predicted structure in a nondifferentiable manner (e.g., via OpenMM), we cannot train our model end to end. This is because we must be able to compute the gradients or construct a forward and a backward component for every layer of our neural network. Adding hydrogens outside of PyTorch would not retain the computation graph necessary for automatic differentiation. In other words, we would not easily be able to compute the derivative of the function that adds hydrogens to the predicted structure.

We developed software in the SidechainNet package to address this issue. Our method adds hydrogens (if needed) to predicted protein structures using only differentiable PyTorch vector operations to maintain differentiability. The software components found in SidechainNet’s SCNProtein class enable building hydrogen-inclusive Cartesian coordinates starting from angle-only representations via a side-chain-parallel method based on natural extension of reference frame (26) or by adding hydrogens to the Cartesian coordinates of existing heavy atoms, also via natural extension of reference frame. Next, our software sets up an OpenMM MD system. Our custom loss function is then used to compute $U\left(X_{H}\right)$ in the forward pass and $-F\left(X_{H}\right)$ in the backward pass by querying these values from OpenMM. Since adding hydrogen atoms via vector operations is differentiable, PyTorch can effectively map the all-atom forces back to the heavy-atom coordinate representation.

Our method adds hydrogens in consistent, predetermined conformations without further optimization. Although this placement may result in nonoptimal hydrogen positions, this does not prevent our system from minimizing the structure’s potential energy.

#### Practical consideration II: Rugged potential energy landscapes

Training a machine learning model using potential energy as a loss function is challenging because protein energy landscapes can be incredibly rugged (27,28). Small changes in side-chain rotamers may cause unrealistic clashes in otherwise perfect structures. Suppose MD FFs evaluate the energy of a protein system with clashes. In that case, the software may return astronomically high energy values (infinite or approaching the maximum value of computational numerical representation). These values reflect the physical impossibility of two atoms occupying the same physical space. This behavior is not frequently observed in MD trajectories because atoms often do not move enough between time steps to overlap or take on unrealistic conformations. However, when training DL models using mini-batches for gradient descent, each subsequent prediction is for a different protein and represents a new MD system. Thus, there are no guarantees that the predicted system will be in a realistic conformation. Furthermore, in a model like AF2, there are several other loss terms in the composite loss function to consider (e.g., frame-aligned point error loss, distogram loss, and masked multiple sequence alignment loss). The raw energy of the predicted system would easily dominate the magnitude of the other loss terms, which we observed as having maximal values of about 1.5 loss units in a trained AF2 model.

To smooth out the energy landscape’s ruggedness and transform the loss’s range into a range that is less likely to dominate other loss terms, we construct a sigmoid function, $\tilde{\sigma }$, by modifying the standard sigmoid function, σ, and applying it directly to the raw energy, *x*.

(3) $$\begin{array}{l} \sigma \left(x\right)={\left(1+e^{-x}\right)}^{-1}\\ \tilde{\sigma }\left(x\right)={\left(\frac{1}{A}+e^{-\frac{\left(Dx+B\right)}{C}}\right)}^{-1}+A+1\\ \sigma^{*}\left(x\right)=\frac{x}{10^{12}}+\tilde{\sigma }\left(x\right) \end{array}$$

Here, *A* = 5, *B* = 10^6^, *C* = 3e+5, and *D* = 5. We heuristically chose several constant scalar parameters to construct a sigmoid function with desirable properties. $\tilde{\sigma }$ has a maximum value of 1, a minimum value of −4, and changes from positive to negative when the energy of a protein structure is about −20,000 kJ/mol. We also selected a loss component weight of 0.01 for the final computed loss value. These values were chosen so that the range of our loss function during training would not differ significantly from the range of values of the other loss components. For example, we reasoned that since AF2 loss components did not become negative during our fine-tuning experiments, we would like our energy-based loss to become negative only after surpassing a low-energy threshold of −20,000 kJ/mol that we associated with energetically favorable protein structures. Allowing our loss function to become negative would enable the model to optimize the predicted structure’s energy further, even when other loss components reached their minimal values. We found that this choice of hyperparameters resulted in more stable training. Due to the computational expense of our experiments, we were unable to evaluate more hyperparameter combinations.

We developed a second modified sigmoid, $\sigma^{*}$, which is a “leaky” version of $\tilde{\sigma }$. $\sigma^{*}$ adds an extremely small fraction of *x* to $\tilde{\sigma }$ (see Fig. 2). This enables constant, nonzero gradients for even extreme energy-loss values. Theoretically, this allows the model to learn from physically implausible structures while still dramatically smoothing the energy-loss landscape. This modification also removes the asymptotic lower bound in $\tilde{\sigma }$ so that models may reach any energetic minimum.

In practice, we found these modifications helpful for training and visualizing energy values since the range of raw energy values of predictions can be too large to plot even on a log-scaled axis.

### Training

#### Models

To measure the effects of our custom loss function, we developed a fork of the OpenFold model that enables training with our data and loss function. We fine-tuned models rather than training from scratch, loading weights provided by the OpenFold authors.

We attempted to match the model training hyperparameters and fine-tuning procedures described in the OpenFold and AF2 papers. However, we did not increase the multiple sequence alignment (MSA) depth or crop size to limit GPU memory consumption. We trained each model using four A100s for about 12 days and achieved an effective batch size of 128 by accumulating gradients 32 times. We could not match the number of GPUs used in the OpenFold paper due to computational resource constraints. To avoid training instability, we used an OpenMM-Loss-specific learning rate schedule that linearly scaled the loss function component weight to its maximum value over 1000 steps. When training with OpenMM-Loss, the added computation accounts for less than 2% total training time.

#### Data

We propose two distinct and mutually compatible ways to train AF2 to predict structures with lower energy and better physical characteristics: training on energetically minimized protein structures and training with our loss function.

We propose training on energetically minimized protein structures for the following reason. Suppose we train AF2 on raw, unminimized PDB structures with our loss function. As mentioned before, structures in the PDB likely do not reflect realistic structures in an aqueous environment. In that case, the accuracy-based (e.g., frame-aligned point error) and energy-based (e.g., OpenMM-Loss) loss function components may push the model in opposing directions, one toward accurately reflecting the PDB structure and another toward predicting a conformation with low energy. Training on protein structures that already exist at a local minimum of our loss function should enable the model to satisfy both objectives simultaneously.

To create this minimized dataset, we used our loss function (without the sigmoid component) and PyTorch to minimize a set of proteins from the SidechainNet dataset. Because our loss function measures protein potential energy via OpenMM and AMBER FFs, minimizing the loss function of each protein within PyTorch is similar to performing energy minimization with OpenMM and AMBER but with the constraint of optimizing in internal coordinate space (bond lengths and angles are fixed to their optimal values in the targeted forcefield). We chose to minimize the energy of our proteins with respect to their bond and torsional angles rather than their atomic coordinates. This theoretically allows models that predict angle representations of proteins to predict their target with perfect accuracy since bond lengths and angles in the minimized set are set to idealized values with respect to the ff15ipq FF. Starting with about 100,000 protein entries from the SidechainNet CASP 12-era (29) dataset, we ended up with about 32,000 entries that 1) did not have gaps in their structures (a requirement for our minimization procedure) and 2) did not deviate more than 5 root-mean-square deviations from their starting structure after minimization. This dataset is called scnmin.

An unminimized version of this dataset containing identical protein entries called scnunmin was retained to evaluate our loss function without minimized protein structures.

We utilized MSAs and template hits generated by the OpenFold authors for training when available (21). We followed the mmseqs2-based (30) procedure suggested by the OpenFold authors to generate the remaining input MSAs and template hits.

We chose a validation set from CAMEO (31) to match the one in the OpenFold paper. We constructed a new test set containing 93 proteins from a 1-year window of CAMEO proteins ending on January 3, 2023. Our validation and test sets were minimized similarly to the training set while retaining their unminimized counterparts. In cases where a protein was rejected from our minimization procedure, we excluded it from both the minimized and unminimized sets. To determine training convergence, we used the minimized version of the validation set when training with scnmin and the unminimized validation set when training with scnunmin.

### Evaluation

It is essential to briefly discuss the distinction between accuracy (e.g., lDDT (32)) and structural quality (e.g., MolProbity score). Methods papers for protein structure prediction have understandably focused on accurately predicting the protein structures available in the PDB. However, as discussed earlier, these structures are not ideal prediction objectives because they are potentially dissimilar from their in vivo counterparts. Additionally, their structures may change significantly in different environments or when bound to other ligands.

Accuracy metrics necessitate a selection of ground-truth structures. Because we trained models to predict either minimized or unminimized structures from the PDB, using accuracy alone to assess our method’s performance would be insufficient. Instead, we propose that a more appropriate assessment of our method would be to compare orthogonal measures of structural quality—such as the MolProbity score or the clash score computed by the MolProbity software suite (33). The MolProbity score for a given structure reflects the crystallographic resolution at which the observed amount of structural irregularities would be expected. For example, a MolProbity score of 0.8 would be the expected score from a structure determined from an X-ray crystallographic experiment with a resolution of 0.8 Å. The clash score measures per-contact clash energy in a protein structure when atoms are unrealistically close to each other (i.e., the number of overlapping atoms). In both cases, lower values are better. For example, protein structures with a resolution (or MolProbity score) of 1 Å or lower are considered very high quality, while values above 3 Å are considered to have significantly less accurate structure information. We assess structural quality metrics before and after AF2-prescribed energy minimization relaxation with OpenMM and AMBER FFs.

We repeated each fine-tuning experiment with three different random seeds to measure the robustness of our method. In the case of AF2, we utilized three different sets of weights reported to have similar performance instead of retraining AF2 from scratch (finetuning_3.pt, finetuning_4.pt, and finetuning_5.pt). In the subsequent plots and analysis, we combined the results from all three seeds for simplicity. However, the trends we observe are consistent across all seeds.

We checkpointed models by their performance on the validation set with respect to the OpenMM-Loss. We computed p values using the one-sided j-test implemented in scipy (34) with respect to the AF2 baseline.

## Results

### OpenMM-loss lowers prediction potential energy while maintaining accuracy

**Figure 3:**
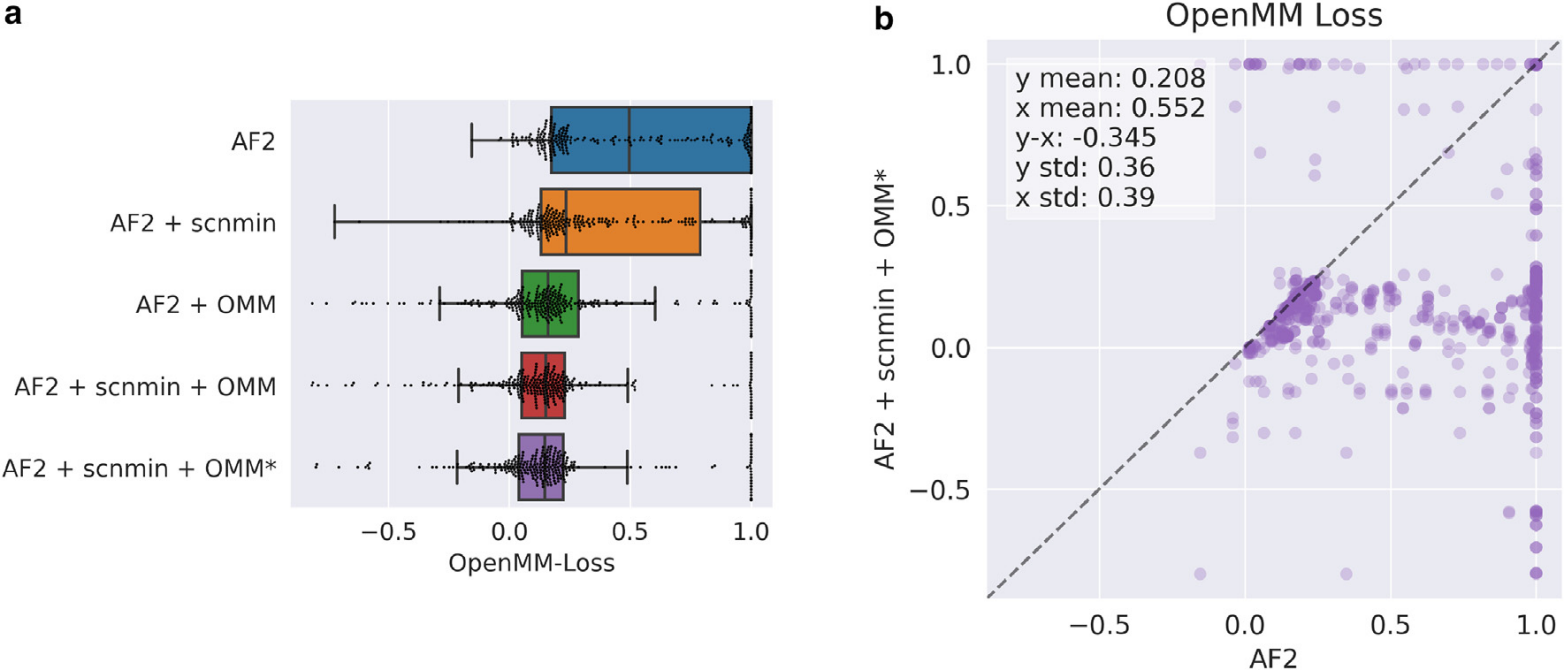
Model prediction potential energy represented by OpenMM-Loss. (*a*) Standard boxplot of distribution of loss values for various methods. Each dot represents a predicted protein structure from the CAMEO test set before relaxation. In (*b*), the x and y positions of each dot correspond to the OpenMM Loss value for the protein when predicted by the methods noted on the x and y axes, respectively. AF2, baseline AlphaFold2 initialized using finetuning_5.pt; + scnmin, fine-tuned on minimized SidechainNet proteins instead of unminimized structures; + OMM, fine-tuned using OpenMM-Loss with $\tilde{\sigma }$ activation; + OMM^∗^, fine-tuned using OpenMM-Loss with $\sigma^{*}$ activation. To see this figure in color, go online.

**Figure 4:**
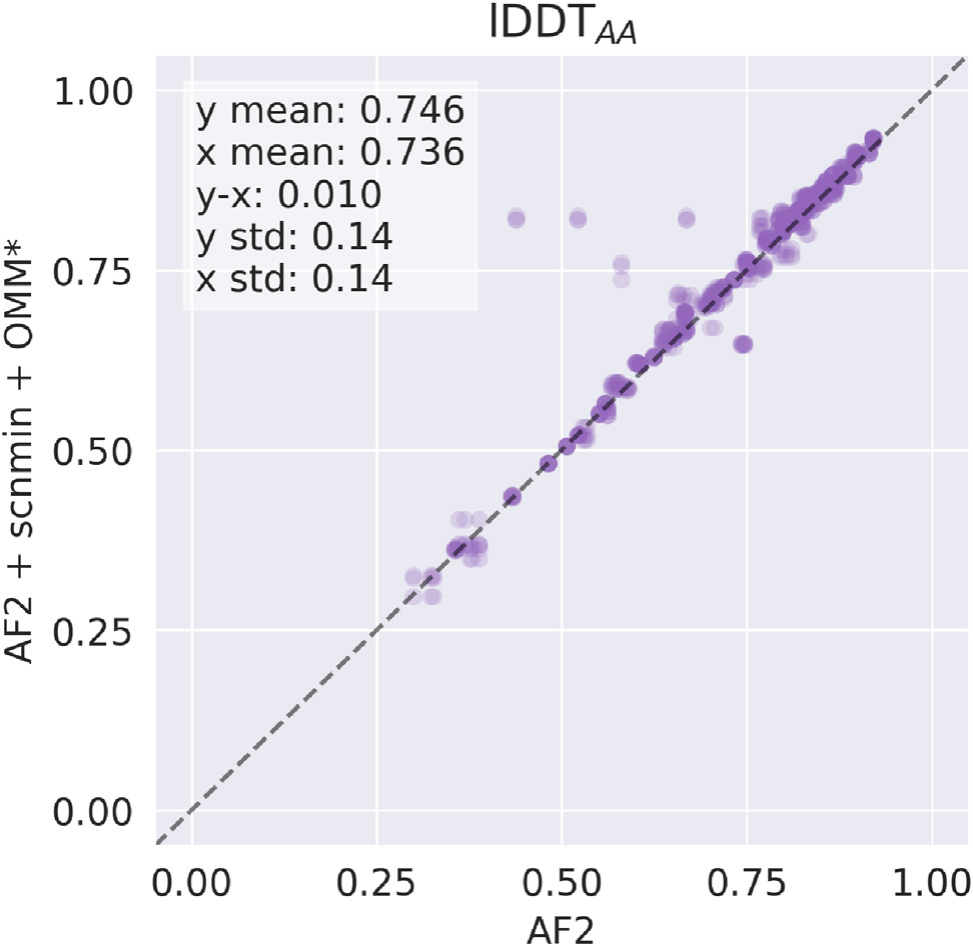
Accuracy between unrelaxed model predictions and the minimized CAMEO test when comparing our method and the AF2 baseline. AF2, baseline AlphaFold2 initialized using finetuning_5.pt; + scnmin, fine-tuned on minimized SidechainNet proteins instead of unminimized structures; + OMM^∗^, fine-tuned using OpenMM-Loss with $\sigma^{*}$ activation. To see this figure in color, go online.

When trained with our loss function, predictions exhibit significantly lower energy-loss values compared to predictions made from AF2 (Fig. 3). Although we do not plot raw energy values (kJ/mol) here, we argue that OpenMM-Loss is an appropriate substitution because it monotonically increases with energy and simplifies the interpretation of extreme values. lDDT_AA_, an all-atom accuracy metric, is not significantly different (p = 0.8) for both methods when evaluated on the energy-minimized CAMEO test set (Fig. 4). We use a simplified implementation of lDDT_AA_ that avoids renaming ambiguous atom names and thus slightly under-reports accuracy.

### OpenMM-loss improves physical properties of predictions before and after relaxation

**Figure 5:**
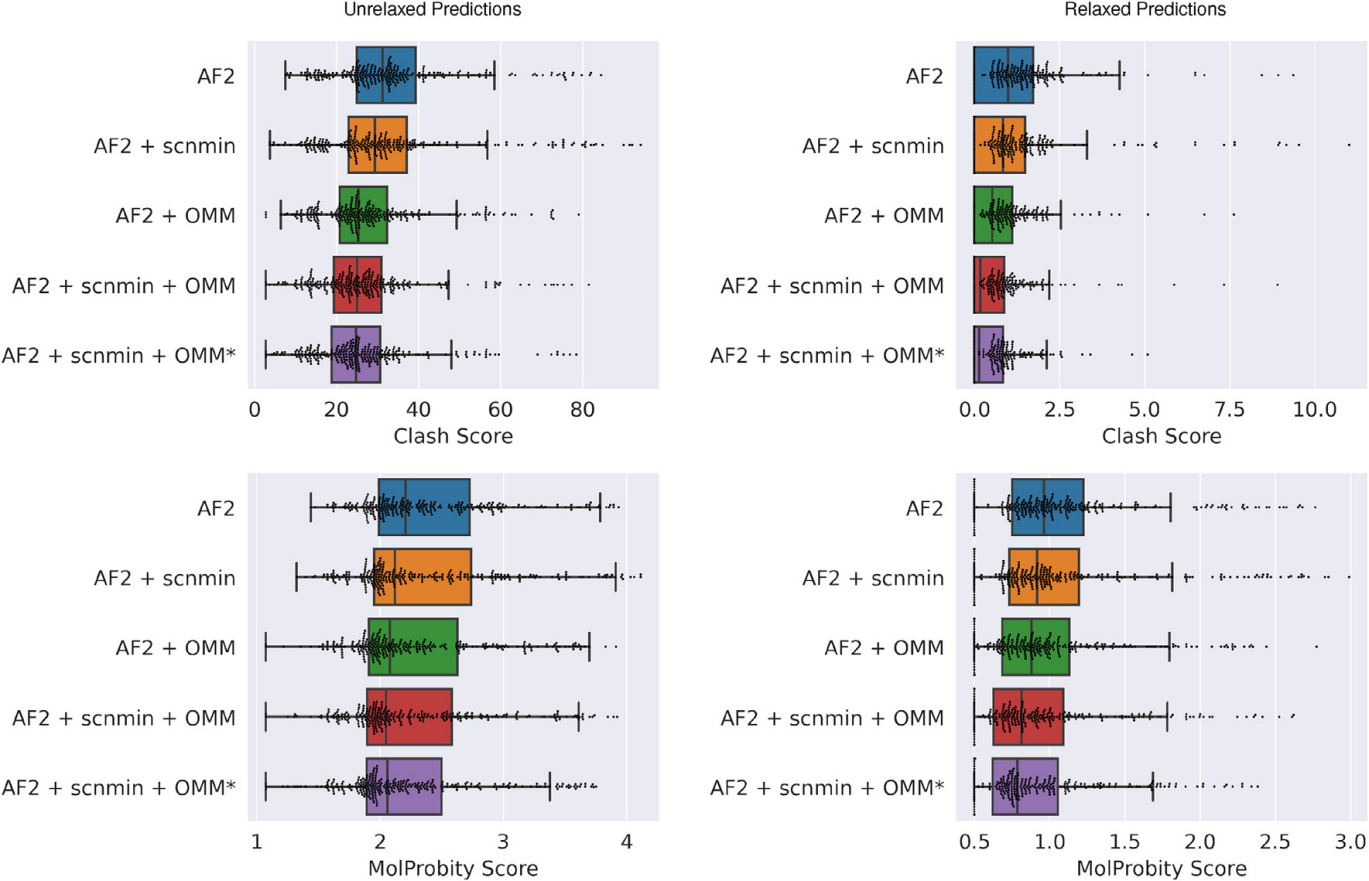
Evaluating the impact of the components of our method on structural quality metrics (clash score and MolProbity score) relative to the AF2 baseline. Standard box plots are shown for each metric and prediction type. Each dot represents a single predicted protein from the CAMEO test set. Predictions are evaluated before and after relaxation (*left and right columns*, respectively). AF2, baseline AlphaFold2 initialized using finetuning_5.pt; + scnmin, fine-tuned on minimized SidechainNet proteins instead of unminimized structures; + OMM, fine-tuned using OpenMM-Loss with $\tilde{\sigma }$ activation; + OMM^∗^, fine-tuned using OpenMM-Loss with $\sigma^{*}$ activation. To see this figure in color, go online.

**Figure 6:**
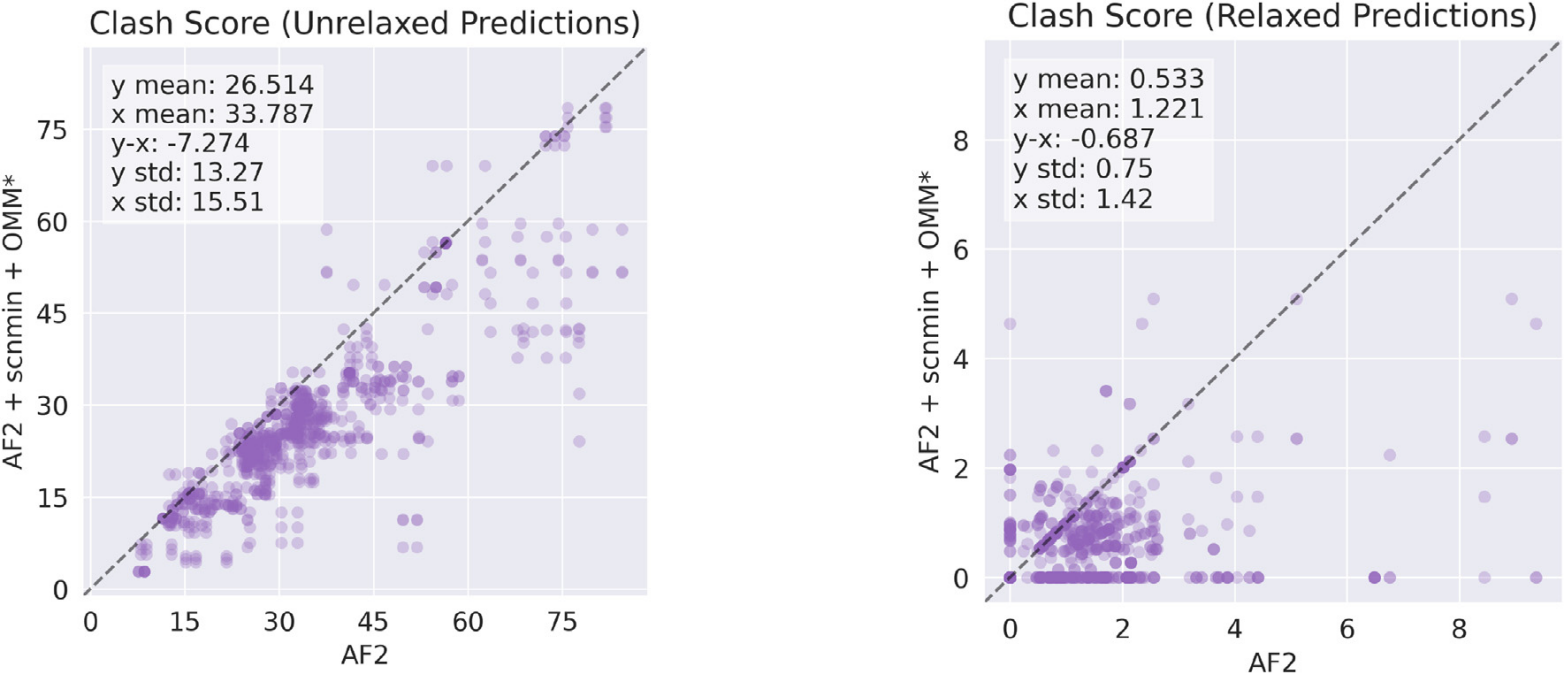
Comparing clash scores for predicted CAMEO test set proteins from one of our trained models (AF2 + scnmin + OMM^∗^) versus the AF2 baseline. Model predictions are evaluated before and after relaxation (*left and right columns*, respectively). AF2, baseline AlphaFold2 initialized using finetuning_5.pt; + scnmin, fine-tuned on minimized SidechainNet proteins instead of unminimized structures; + OMM^∗^, fine-tuned using OpenMM-Loss with $\sigma^{*}$ activation. To see this figure in color, go online.

Our method’s predictions have more favorable MolProbity scores and clash scores on average than predictions from AF2 (Figs. 5 and 6; Table 1). Our method also has significantly more predictions with the lowest possible values of either metric (0.5 and 0, respectively). We observe a trend from top to bottom in Fig. 5 of improving structure quality as we add the components of our method (training on minimized structures, scnmin, and training with our custom loss, OMM/OMM^∗^).

**Table 1:** Mean structure quality metrics of structure predictions before and after relaxation

Model	MolProbity score (p value)		Clash score (p value)	
	Unrelaxed	Relaxed	Unrelaxed	Relaxed
AF2	2.389	1.077	33.787	1.221
AF2 + scnmin	2.375 (4e−01)	1.087 (6e−01)	32.910 (3e−01)	1.186 (4e−01)
AF2 + OMM	2.285 (2e−02)	0.986 (1e−02)	28.156 (3e−06)	0.744 (3e−06)
AF2 + scnmin + OMM	2.271 (8e−03)	0.945 (8e−04)	27.143 (6e−08)	0.598 (2e−09)
AF2 + scnmin + OMM^∗^	**2.244** (1e−03)	**0.915** (3e−05)	**26.514** (2e−09)	**0.533** (2e−12)

Computed p values reflect the significance of being lower than the AF2 baseline. The bold indicates the best value.

**Figure 7:**
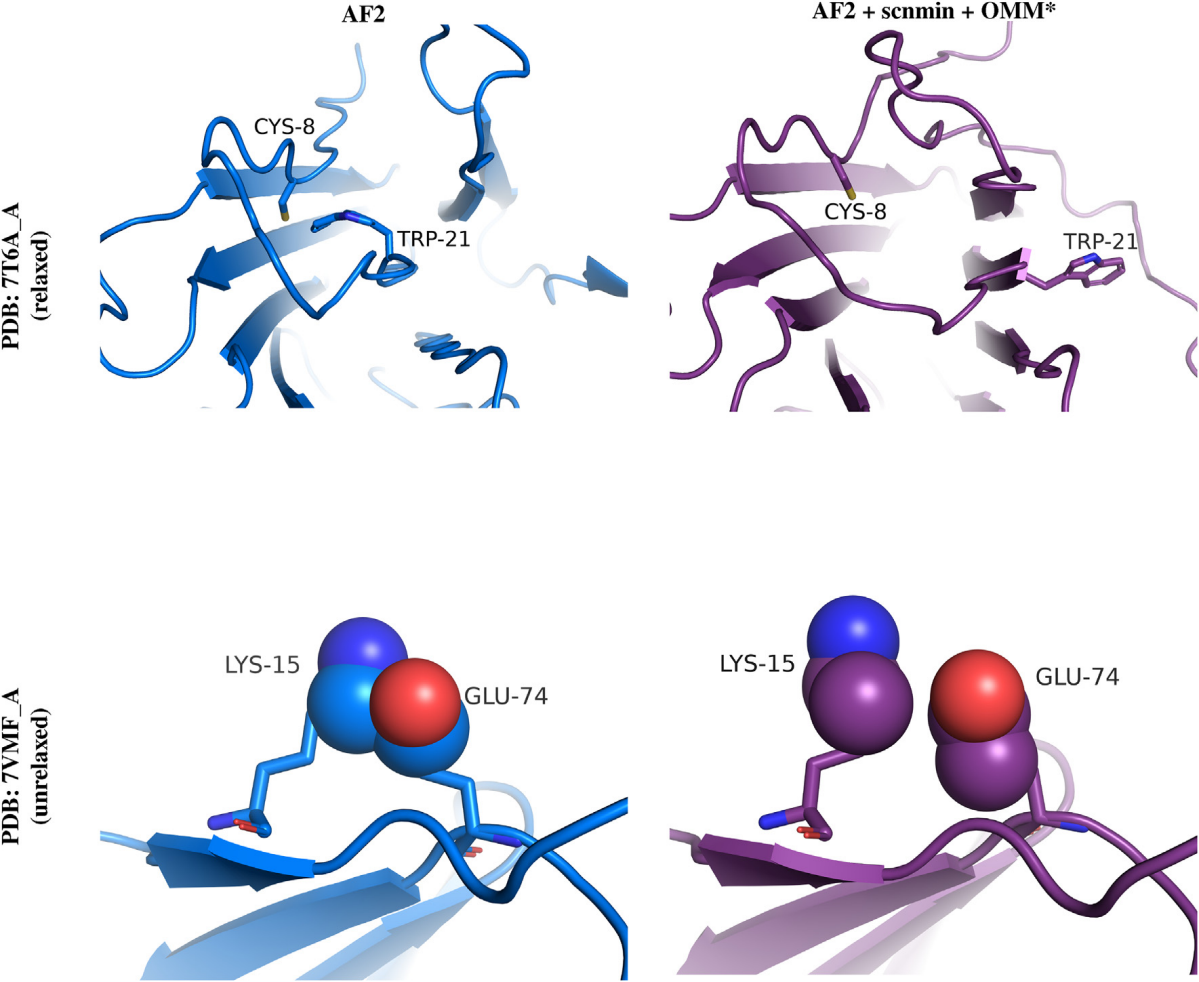
Protein structure prediction examples from the AF2 baseline (*left*, *blue*) versus our AF2 + scnmin + OMM^∗^ method (*right*, *purple*). 7T6A_A structures (*top*) are compared after AF2’s relaxation procedure, while 7VMF_A’s structures (*bottom*) are unrelaxed. Our method resolves overlapping atoms in both sets of structures and improves the TRP-21 geometry in 7T6A_A from a bent to flat ring structure. To see this figure in color, go online.

It is worth noting that training on minimized structures (scnmin) alone does not significantly improve accuracy or structural quality metrics despite lowering the potential energy of predicted structures on average. In contrast, our loss function significantly improves structural quality relative to the AF2 baseline. The best improvement is seen by combining these two training strategies. Illustrative examples where our models have reduced clashes and improved structure geometry can be seen in Fig. 7.

The accuracy of our models between unrelaxed structures and the minimized CAMEO test set labels is shown in Table 2. Our objective is to produce more realistic predictions while not necessarily reproducing structures from the PDB, so we do not place a significant emphasis on accuracy values. It should be noted that direct comparison of accuracy metrics from the minimized CAMEO test set against the baseline AF2 has a caveat: AF2 is not trained to predict minimized structures, whereas all our methods are. Similar to our results regarding structural quality, we find that fine-tuning on minimized data and with OpenMM-Loss has a larger increase in accuracy than utilizing either one of these approaches individually.

**Table 2:** Structure prediction accuracy between unrelaxed predictions and the minimized CAMEO test set structures

Model	lDDT_*AA*_	lDDT_*Cα*_	TM score
AF2	0.736	0.818	0.671
AF2 + scnmin	0.744	0.826	0.679
AF2 + OMM	0.737	0.816	0.668
AF2 + scnmin + OMM	0.745	0.829	**0.682**
AF2 + scnmin + OMM^∗^	**0.746**	**0.830**	**0.682**

### OpenMM-loss results in lower energy structures for MD

**Figure 8:**
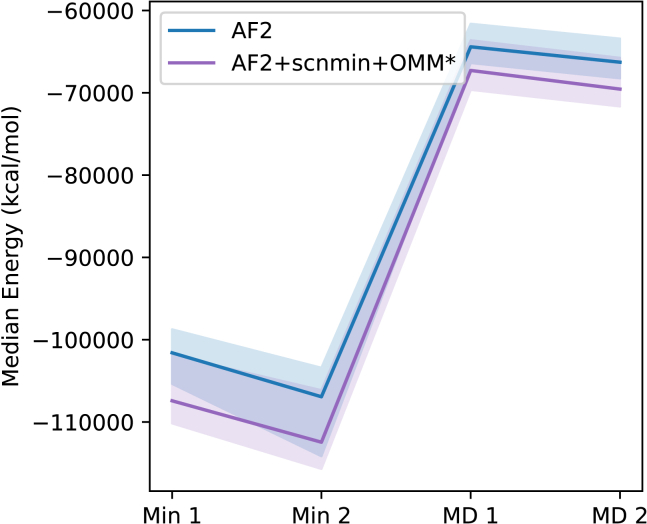
Median energy of structures as they are prepared for MD. The final energy of each minimization stage and the average energy of each MD equilibration are computed. The median across the 93 structure test set is shown with a 50% confidence interval. To see this figure in color, go online.

In order to evaluate the effect of using our method in a downstream task of structure prediction such as MD, we performed an initial equilibration of each of the predicted structures in our test set using Amber 2020 (35). Each system was solvated and neutralized using tleap and then subjected to two rounds of energy minimization, the first (Min 1) with restraints on the protein and the second (Min 2) with no restraints. Minimization was followed by 100 ps constant temperature and volume (NTV) MD where the protein was restrained and the system was heated from 0 to 300 K (MD 1), and then a further 100 ps unrestrained constant temperature and pressure (NTP) simulation at 300 K and 1 atm (MD 2). As with OpenMM-Loss, the AMBER ff15ipq FF (25) with TIP3P water was used due to its ability to reproduce NMR experimental observables (36). As shown in Fig. 8, at each step of this process, the median energy of the test set was lower when using a structure generated from an OpenMM-Loss model compared to an AF2 model trained without OpenMM-Loss.

## Discussion

If protein structure predictions are to become a valuable and practical tool for downstream tasks, the scientific community requires two things. First, we must improve accuracy at the side-chain level to avoid subtle changes in protein structure that have outsized impacts on tasks like molecular docking. Second, we require new paradigms to incorporate theoretical physical principles. By utilizing physical principles during training, it may be possible to continue the steady march toward improved accuracy while enforcing that predicted structures, at a minimum, pass the basic test of physical plausibility.

In our attempt to address these goals, we have shown that AF2 can be trained to predict structures with lower energy and fewer clashes without a significant change in accuracy. Accuracy in this experiment is difficult to define because we have compared models trained with minimized and unminimized labels. Still, we provide a framework for improving other important metrics related to the structural quality of protein structure predictions. We also hope to instigate a more thorough discussion on the relationship between structural accuracy (e.g., lDDT) and quality (e.g., MolProbity) metrics, which are not always congruent.

A notable contribution of this work is that at inference time, there is no difference in computational cost between a model trained with our loss function and the same model trained without our loss function. Improvements in structural quality are essentially free if one uses the parameters from our fine-tuned version of AF2 instead of the standard weights. Our method has not removed the necessity for relaxation or further refinement of predicted structures, but it is able to produce structures with lower energy and fewer clashes, making its predictions more realistic and attractive for downstream tasks. Though it is true that our loss function adds some computational complexity when training, we found that calculating the OpenMM-Loss made up less than 2% of the total training time.

Further refinement with MD or other methods (9) is possible and orthogonal to our method. We have shown that structures predicted via our method have consistently lower energy when utilized for MD. Additionally, if new, higher-accuracy models or architectures are developed for all-atom protein structure prediction, our work suggests that repeating our training procedure may similarly improve the structural quality of such methods.

Another interesting result from our experiments suggests that the best improvements in structural quality come from training on minimized protein structures in addition to utilizing OpenMM-Loss. Future work must consider whether or not to include this minimization step depending on available computational resources. The minimized protein structures produced for this paper are accessible via our public code repositories (see supporting material).

Finally, a major limiting factor of current protein structure prediction methods is the lack of ligand information at training or evaluation time. A loss function like ours could help models better discriminate between favorable and unfavorable conformations of proteins and protein-ligand complexes by evaluating the potential energy of predicted systems during training. A model capable of understanding even a modest level of chemical interaction principles (for example, understanding how to orient hydrogen donors or acceptors of a ligand within a binding pocket or identifying and rejecting systems with overlapping atoms) may have a significant advantage over models agnostic to such principles. Though our framework only supports all-atom protein representations at present, future work may study the impact of using an energy-based loss function for models that include protein-ligand, ligand-only, or coarse-grained systems.

## Supporting material

Our fork of OpenFold that enables training with OpenMM-Loss can be found at https://github.com/jonathanking/openfold under a permissive Apache 2.0 License. Our custom loss function, along with supporting software and datasets, can be found at https://github.com/jonathanking/sidechainnet under a permissive BSD-3-Clause license.

## Author contributions

J.E.K. and D.R.K. designed the project’s goals and scope. J.E.K. implemented the methods, carried out the experiments, performed the primary analysis, and wrote the manuscript with guidance from D.R.K. D.R.K. analyzed The method for use in MD simulations.
